# Band Gap Engineering in Ultimately Thin Slabs of CdTe with Different Layer Stackings

**DOI:** 10.3390/ma16237494

**Published:** 2023-12-04

**Authors:** Vladimir G. Kuznetsov, Anton A. Gavrikov, Alexander V. Kolobov

**Affiliations:** 1 Ioffe Institute, 26 Polytechnicheskaya Str., 194021 St. Petersburg, Russia; vladimir.kuznetsov@mail.ioffe.ru; 2 Center for Computational Materials Science, Institute of Physics, Herzen State Pedagogical University of Russia, 48 Moika Emb., 191186 St. Petersburg, Russia; 3 Department of Physical Electronics, Institute of Physics, Herzen State Pedagogical University of Russia, 48 Moika Emb., 191186 St. Petersburg, Russia

**Keywords:** 2D chalcogenides, CdTe, electronic structure, band gap thickness dependence, DFT

## Abstract

Ultrathin solid slabs often have properties different from those of the bulk phase. This effect can be observed both in traditional three-dimensional materials and in van der Waals (vdW) solids in the few monolayer limit. In the present work, the band gap variation of the CdTe slabs, induced by their thickness, was studied by the density functional theory (DFT) method for the sphalerite (zinc-blende) phase and for the recently proposed inverted phase. The sphalerite phase has the Te–Cd–Te–Cd atomic plane sequence, while in the inverted phase Cd atoms are sandwiched by Te planes forming vdW blocks with the sequence Te–Cd–Cd–Te. Based on these building blocks, a bulk vdW CdTe crystal was built, whose thermodynamical stability was verified by DFT calculations. Band structures and partial densities of states for sphalerite and inverted phases were calculated. It was demonstrated for both phases that using slabs with a thickness of one to several monolayers for sphalerite phase (vdW blocks for inverted phase), structures with band gaps varying in a wide range can be obtained. The presented results allow us to argue that ultrathin CdTe can be a promising electronic material.

## 1. Introduction

Reducing the thickness of bulk solids to the limit of a few monolayers (ML) often reveals new properties of a material. Most intriguing effects appear in quasi-two-dimensional (2D) layered materials, in which monolayers of covalently bonded atoms are held together by weak van der Waals (vdW) forces. As an example one can consider the transition metal dichalcogenide semiconductor MoS_2_, one of the most studied 2D materials with an indirect band gap of 1.29 eV for the bulk phase, which changes to a direct gap of 1.90 eV for a single monolayer [1], making MoS_2_ and its analogues promising materials for optoelectronics. Likewise, ultrathin 2D InSe possesses a large band gap tunability from 2.39 eV for a single monolayer to 1.24 eV and 1.18 eV for 10 monolayers of two distinct structures β-InSe and γ-InSe (with different stacking patterns) respectively [2]. Such strong dependence of the band gap value on the layer thickness is very attractive for applications in the field of novel devices of solid state electronics including flexible and transparent electronics.

It should be noted that in most quasi-2D layered semiconductors monolayers of covalently bonded atoms are terminated by chalcogen planes between which van der Waals forces are established, which makes chalcogenides a very special class of solids with a strong potential to form van der Waals allotropes.

A strong thickness dependence of material’s structure and properties can also be observed in ultrathin slabs of conventional three-dimensional (3D) semiconductors. Thus GaN, which has the wurtzite structure in the bulk form, takes a planar graphene-like geometry when thinned to one monolayer, and further changes to a haeckelite structure for slabs with thicknesses from 2 to 4 monolayers [3,4]. A few-monolayer thick haeckelite GaN slabs have a direct band gap. Application of strain allows one to change the optical band gap of GaN slabs in a wide range [5].

Cadmium telluride (CdTe) is an important material for modern solid state technologies such as light emitting diodes [6], solar cells and photovoltaics [7,8]. Cadmium chalcogenides in the bulk form can crystallize in two phases, the cubic zinc-blende structure and/or hexagonal wurtzite structure. Their slabs are also intensively studied due to their optical properties varying with the thickness of the material [9,10]. They contain chalcogen species which as mentioned above, are necessary to realize layered structures. Recently, the electronic structure of *zb*-2ML CdTe with the Cd-Te-Cd-Te atomic-plane sequence and the so-called inverted structure (*inv*-2ML) with Cd atoms sandwiched between Te planes (the Te–Cd–Cd–Te atomic-plane sequence) were studied by some of the present authors using the density functional theory (DFT) method within generalized gradient approximation (GGA) with PBEsol exchange-correlation (XC) functional [11]. It was demonstrated that both structures were thermodynamically stable while the inverted structure was energetically more favourable. Thereby the hypothesis about the polymorphism of 2D CdTe slabs was confirmed. This result can be readily understood considering that (i) the Cd/Te plane inversion reduces the dipole moment of the structure surfaces and (ii) is in full agreement with the claim that chalcogen termination of the surface was energetically more favourable [12]. It was also noted that fabrication of CdTe nanoplatelets of 5 MLs or greater and the thickness control of CdTe nanoplatelets remained challenging [9]. At the same time, in [13] the authors demonstrated the formation of atomically flat quasi-two-dimensional colloidal CdSe, CdS and CdTe nanoplatelets with well-defined thicknesses ranging from 4 to 11 monolayers. The authors note that these nanoplatelets possess the electronic properties of two-dimensional quantum wells formed by molecular beam epitaxy and discuss the thickness dependence of their absorption and emission spectra.

A detailed review of experimental and theoretical works devoted to the study of two-dimensional A^II^B^VI^ semiconductors is given by Diroll et al. [9]. In particular, it was reported that the minimal thickness of cadmium chalcogenides slabs (also referred in literature as nanosheets or nanoplatelets) experimentally obtained to date by the colloidal synthesis method corresponds to the thickness of a 2ML slab. We note in passing that experimentally obtained slabs are characterized [9] by the presence of an additional plane of metal atoms in the slab, e.g., 2ML nanoplatelet of CdTe has the Cd–Te–Cd–Te–Cd atomic-plane sequence in contrast to the 2ML slab with the Cd–Te–Cd–Te atomic-plane sequence obtained in theoretical calculations [11]. It is worth mentioning here that these slabs were obtained in colloid solutions and in reality contain ligands on their surfaces, i.e., their existence does not contradict the proposal that Cd atoms must be located inside the slab.

In [10] the thickness dependence of the band structure for 2D CdSe slabs was studied by the DFT method where a decrease of the band gap value with increasing thickness of CdSe from 1ML to 6ML was shown. In addition, the dependence of the exciton transition energy on the thickness of CdTe slabs was studied experimentally for CdTe *n*ML (*n* = 5–9) slabs and was found to be linear [14]. It was also demonstrated that the exciton transition energy decreases with increasing slab thickness. Thus it can be argued that changes in the band structure are associated with changes the thickness of 3D materials. Therefore theoretical studies of band gap variation are of special interest for the development of solid state devices.

The experimental band gap value for bulk CdTe with the zinc-blende structure (*zb*-bulk) is about of 1.60 eV [15]. The best value of the DFT band gap for *zb*-bulk CdTe was obtained with the semi-local Tran-Blaha XC potential [16] and was found to be 1.56 eV [17]. However, as regards the type and the value of the band gap for a single two-dimensional CdTe covalently bonded layer, in what follows referred to as a monolayer (*zb*-1ML), different theoretical articles contain contradictory results [18,19,20]. We provide a review and discussion of these theoretical results below in Section 3.

In the present work, we carried out a detailed DFT study, both without spin-orbit coupling (SOC) and with it, of the band gap variation along with the densities of states (DOS) for 2D CdTe slabs in the zinc-blende phase with a thickness of *n*ML (*n* = 1–4) and in the inverted phase formed by inverted blocks referred to hereafter as van der Waals monolayers (1 vdW = *inv*-2ML) with a thickness of *n*vdW (*n* = 1–3). Later from such vdW blocks we constructed a bulk CdTe vdW crystal and calculated its band structure. Based on the band structure calculations of CdTe slabs with different thickness, we obtained the band gap thickness dependencies. Then we compared them with calculated band gap values of the bulk *zb*-CdTe crystal and bulk vdW CdTe crystal. In addition, we calculated partial densities of states (PDOS) for the bulk CdTe and monolayers of CdTe both in the zinc-blende phase and in the inverted phase. Based on the obtained results we argue that CdTe monolayers in both phases are suitable for devices requiring band gap tunability.

## 2. Materials and Methods

CdTe *n*ML (*n* = 1–4) slabs with *zb* structure were built from a bulk crystal by cleaving along the (111) surface. The *zb* structure is characterized by alternating –Cd–Te–Cd–Te– atomic planes. The *inv*-2ML slab has an inverted layer sequence Te–Cd–Cd–Te, where the outer planes are formed by chalcogen atoms, whereas the metal atoms are located inside the block. For details of the *inv*-2ML block generation see [11]. From such blocks a bulk vdW crystal was built.

**Figure 1:**
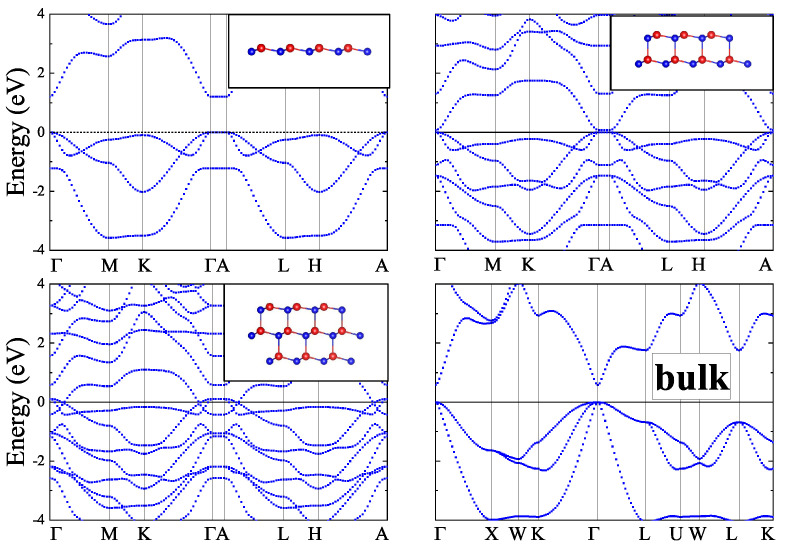
Band structures of CdTe *zb*-*n*ML (*n* = 1−3). The lower righthand panel shows the band structure of the bulk crystal.

**Figure 2:**
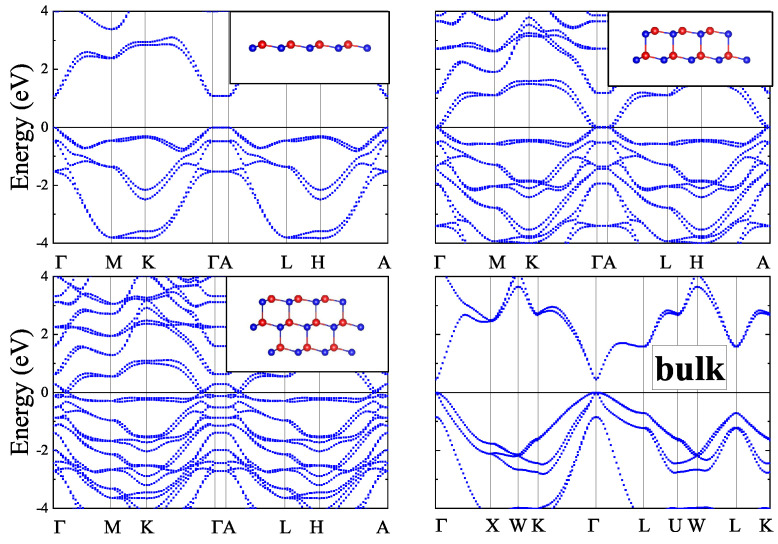
Band structures for *zb*-*n*ML (*n* = 1−3) and bulk *zb*-CdTe with SOC.

**Figure 3:**
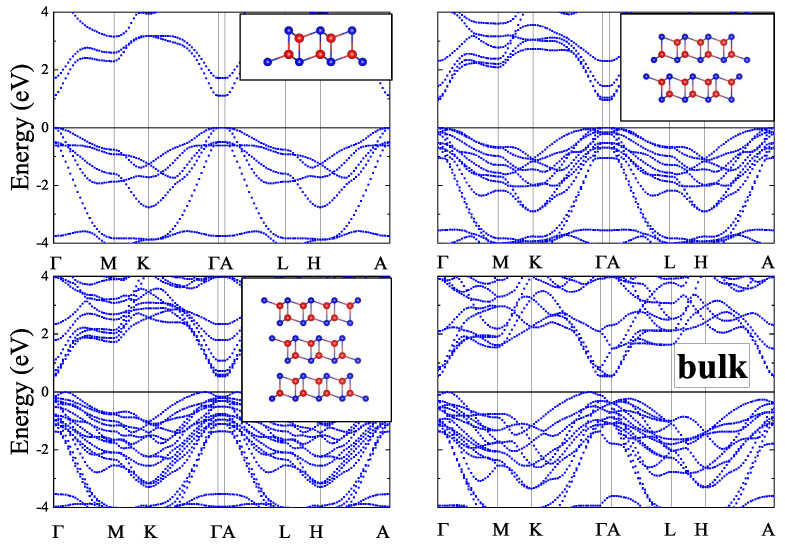
Band structures of *n*vdW (*n* = 1−3) blocks and bulk vdW CdTe crystal.

**Figure 4:**
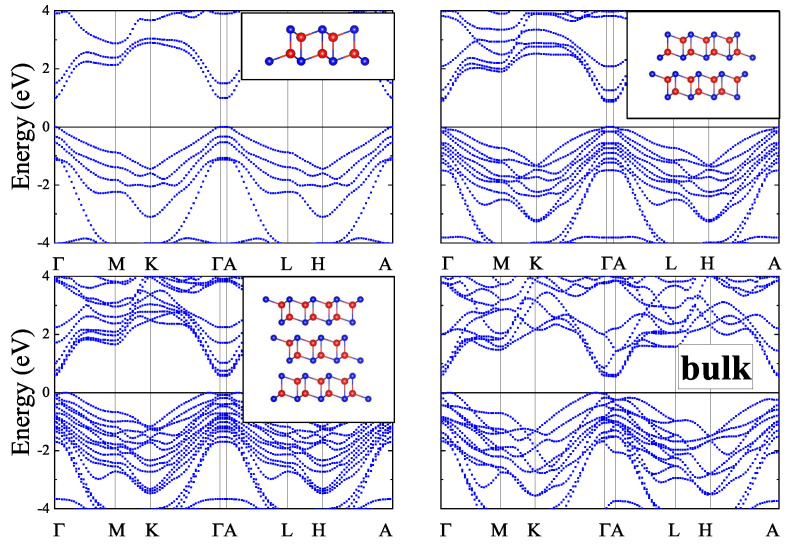
Band structures of *n*vdW (*n* = 1−3) blocks and bulk vdW CdTe crystal with SOC.

In the present work, 2D *zb*-CdTe slabs with a thickness of 1–4 monolayers and *inv*-CdTe slabs with thicknesses of 1–3 vdW blocks were studied in-silico along with the bulk *zb*- and vdW-CdTe crystals. The structures of the slabs, visualized using the VESTA 3 software [21], are shown as insets in Figure 1, Figure 2, Figure 3 and Figure 4 (in the upper right corner).

Simulations were carried out using the DFT method within the GGA approach with the XC PBE [22] and PBEsol [23] functionals and vdW correction term in the form of Grimme-D2 [24] as implemented in the plane-wave pseudopotential-based code CASTEP [25,26]. To describe the electron-ion interactions scalar-relativistic (SR) optimized norm-conserving Vanderbilt (ONCV) pseudopotentials [27] from the Schlipf–Gygi pseudopotential library [28] (issue 6 February 2020) were used in calculations without SOC, and fully relativistic (FR) norm-conserving (J-dependent) pseudopotentials from the CASTEP library in calculations with SOC, wherein the 4s${}^{2}$4p${}^{6}$4d${}^{10}$5s${}^{2}$ electrons of the Cd atoms for both types of pseudopotentials (SR and FR) and the 4d${}^{10}$5s${}^{2}$5p${}^{4}$ electrons for SR pseudopotential (5s${}^{2}$5p${}^{4}$ electrons for FR pseudopotential) of the Te atoms were assigned to the valence space.

Full geometry relaxation for different CdTe slabs was accomplished using the two-point steepest descent (TPSD) algorithm [29]. This algorithm is specifically faster and more efficient when cell optimization with supplied constraints is requested, compared with the generally recommended Broyden–Fletcher–Goldfarb–Shanno (BFGS) optimizer [30], which is significant when optimizing the cell geometry of 2D *n*ML and *n*vdW slabs of CdTe with a fixed value of the vacuum gap in the periodic strucure.

The relaxation was carried out until the maximum values of the energy difference per atom, the Hellmann–Feynman forces on the atoms, and all the stress components became less than 2 × 10${}^{-7}$ eV atom${}^{-1}$, 5 × 10${}^{-3}$ eV Å${}^{-1}$, and 1 × 10${}^{-2}$ GPa, respectively. A tolerance for convergence of the self-consistent field energy was taken equal to 5 × 10${}^{-8}$ eV atom${}^{-1}$. The kinetic energy cutoff value of 1200 eV was taken determined by the use of ONCV pseudopotentials in order to achieve the energy convergence of a few meV when using of ONCV pseudopotentials. The *k*-point meshes for different *n*ML and *n*vdW slabs of CdTe were chosen to ensure that the respective maximum *k*-point spacings are roughly the same for different slabs and equal approximately to 0.035 Å${}^{-1}$, which ensures a comparable accuracy of the calculations for all structures.

The phonon dispersion spectrum of the bulk vdW CdTe crystal was calculated for the equilibrium geometry using the linear response approach or the density functional perturbation theory (DFPT) [31] and its CASTEP implementation within the plane-wave pseudopotential formalism [32].

## 3. Results and Discussion

**Figure 5:**
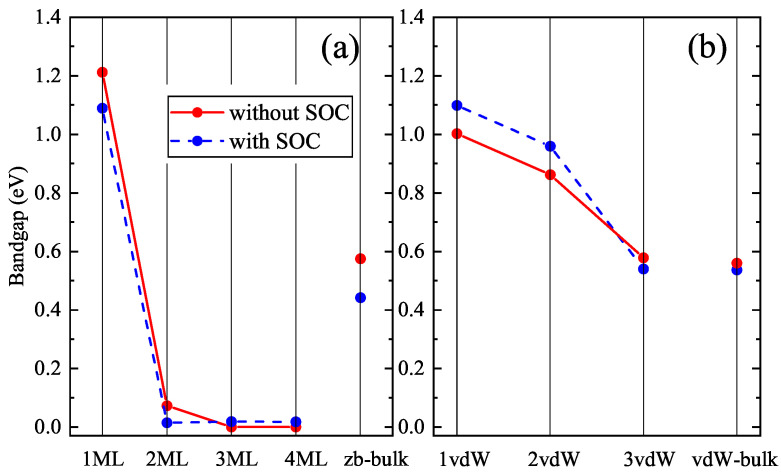
Thickness dependence of the band gap value of (**a**) *zb*-CdTe and (**b**) *inv*-CdTe.

**Figure A1:**
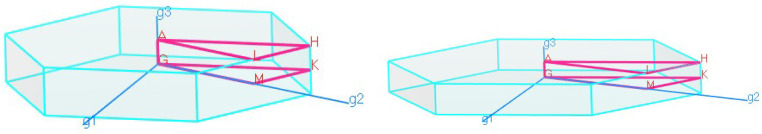
Brillouin zone of *zb*-CdTe slabs (**left panel**) and of vdW CdTe slabs (**right panel**).

**Figure A2:**
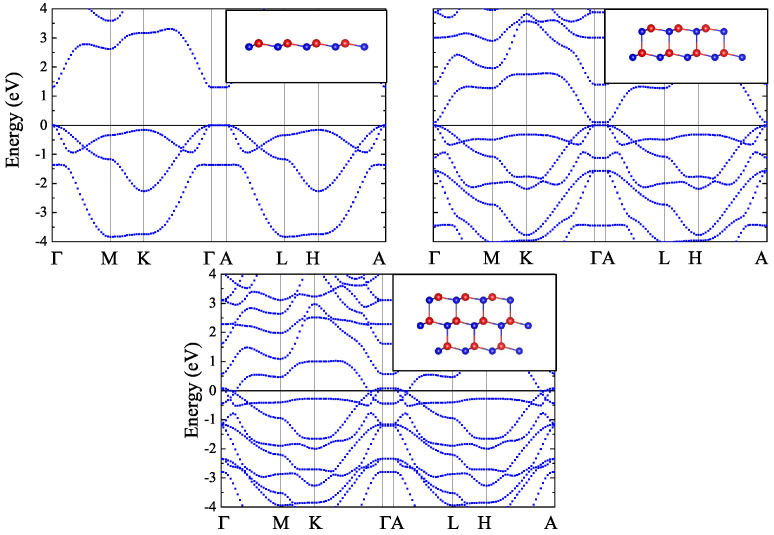
PBEsol band structures for *zb*-*n*ML (*n* = 1−3) without SOC.

**Figure A3:**
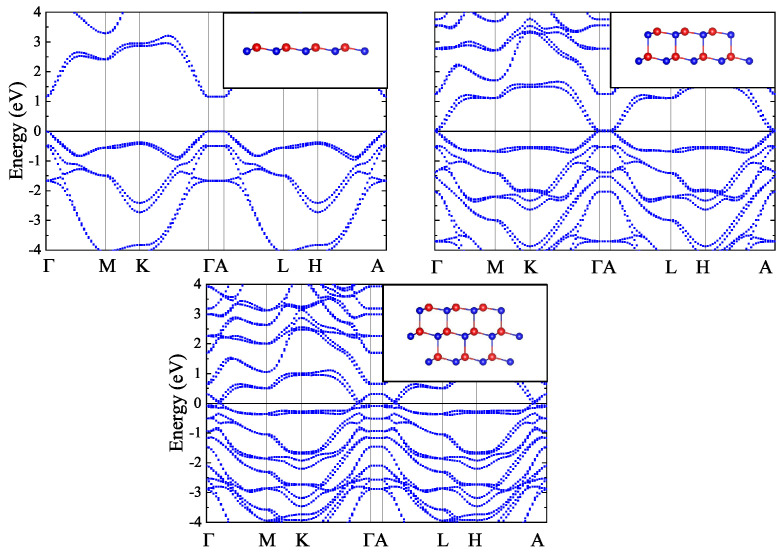
PBEsol band structures for *zb*-*n*ML (*n* = 1−3) with SOC.

Based on the results of Ref. [11], we performed a detailed study of the thickness dependence of ultrathin layers of CdTe in both *zb*- and *inv*- phases. The band structures of *zb*-nML (*n* = 1–4) slabs having space group P3m1 (Schoenflies name C${}_{3v}^{1}$) and that of *n*vdW (*n* = 1–3) slabs having space group P$\bar{3}$m1 (Schoenflies name D${}_{3d}^{3}$) were calculated along the *k*-path Γ–M–K–Γ–A–L–H–A in the irreducible Brillouin zone (see Figure A1) and are shown for the case *n* = 1–3 in Figure 1 and Figure 2. One can see from Figure 1 that the band gap value is close to zero for *zb*-2ML, *zb*-3ML, *zb*-4ML slabs, while for the *zb*-1ML slab the band gap drastically increased to a value of 1.2 eV, whereas for bulk *zb*-CdTe the obtained DFT band gap is just above 0.5 eV. The *zb*-1ML CdTe slab is a direct-gap semiconductor with the minimal band gap value located at the Γ-point. Inclusion of SOC does not lead to a significant change in the band structures for thin layers and for the bulk *zb*-CdTe as well (see Figure 5). The same results were obtained with PBEsol XC-functional (see Figure A2 and Figure A3 in Appendix A).

Below we make a brief review of the previously reported band structures calculations and compare them with our results. As mentioned in Section 1, the band gap of the *zb*-bulk CdTe calculated in the paper [17] using DFT within full-potential linearized augmented plane wave (FP-LAPW) method with the Tran-Blaha semilocal XC potential [16] as implemented in WIEN2k code [33], is equal to 1.56 eV, which is close to the experimental value. This result agrees well with our values obtained with PBE and PBEsol XC-unctionals (0.575 eV and 0.680 eV respectively) for bulk *zb*-CdTe, taking into account that the semilocal XC-functionals often underestimate the band gap by about 50%.

While the electronic structure of the bulk *zb*-CdTe has been studied by the DFT method quite thoroughly, results for CdTe monolayers are controversial. Thus, in [18], where the electronic structure of *zb*-1ML was studied by DFT FP-LAPW method, it was found that a CdTe monolayer is a semiconductor with an indirect-gap value of 1.022 eV calculated with the Tran-Blaha semilocal XC-potential [16] and of 0.773 eV calculated with PBEsol [23] XC-functional. This result disagrees with the direct band gap value of 1.303 eV calculated with PBEsol XC-functional [23] obtained in the present study. The difference of almost twice in the values of the PBEsol band gap calculated in our study and in paper [18] cannot be due to the additional approximation introduced by the use of the high-quality ONCV-pseudopotentials [27] in the present calculations.

As for 2ML CdTe slabs, apart from our DFT calculations of *zb*-2ML and *inv*-2ML structures, we are aware of only two papers [19,20]. One of them is devoted to the α-2ML slab, and the other one to the *inv*-2ML slab. The α-phase of CdTe was proposed in [19], where 2ML slab of α-CdTe (α-2ML) having space group *P4/nmm*, was reported as a dynamically stable phase in which a planar layer of the square Cd lattice is bonded to Te atoms tetrahedrally. It was shown through DFT simulations that α-2ML is a direct band gap semiconductor with a gap value of 1.95 eV. The band gap value was calculated using Projected Augmented Wave potentials [34] and Heyd-Scuseria-Ernzerhof (HSE06) hybrid XC-functional [35] with D2-Grimme vdW correction [24] taking into account SOC as implemented in the VASP code [36,37]. It was concluded that α-2ML CdTe is a promising material for nanoscale optoelectronic applications. Later in paper [11] a comparative analysis of the relative energies (per formula unit) for *zb*-2ML, *inv*-2ML and α-2ML CdTe slabs was performed and it has been shown that the α-2ML slab is energetically higher by 87 meV (per formula unit) than the *inv*-2ML slab.

The second article [20] concerns a DFT study of the *inv*-2ML (1vdW) CdTe slab, whose electronic structure was calculated with the HSE06 hybrid XC-functional [35] and the PAW [34] potential for describing electron-ion interaction as implemented in the VASP code [36]. It has been shown that *inv*-2ML CdTe is a direct gap semiconductor with the Γ-point band gap value of 1.82 eV. This result correlates well with band gap values of 1.099 eV (without SOC) and 1.002 eV (with SOC) obtained in our DFT calculations with PBE XC-functional [22] and ONCV pseudopotentials [27], taking into account that the semilocal XC-functionals often underestimate the band gap by about 50%.

As for the *zb*-*n*ML (*n* = 2–4) and *n*vdW (*n* = 2, 3) CdTe slabs we didn’t find any band gap values in literature. The present DFT calculations of the *zb*-nML band structures displayed in Figure 1 give for the PBE band gaps a value of 0.073 eV for *n* = 2 and near zero values for *n* = 3, 4 respectively. The inclusion of SOC slightly opens the gap for *n* = 3, 4 up to values of 0.019 and 0.018 eV, respectively, and reduces the value of the band gap down to 0.015 eV for *n* = 2. The corresponding band structures with SOC are shown in Figure 2.

When calculating the band structure of the *n*vdW CdTe slabs we included the vdW correction in the form D2-Grimme [24] to the PBE XC-functional [22]. The calculated *n*vdW band structures presented in Figure 3 give for the PBE+D2 band gaps the value of 0.959 eV for *n* = 2 and 0.540 eV for *n* = 3 respectively. The inclusion of SOC reduces the band gap value down to 0.862 eV for *n* = 2 and increases the band gap value up to 0.578 eV for *n* = 3. The corresponding band structures with SOC are shown in Figure 4. Similar results are obtained for bulk crystals.

**Figure 6:**
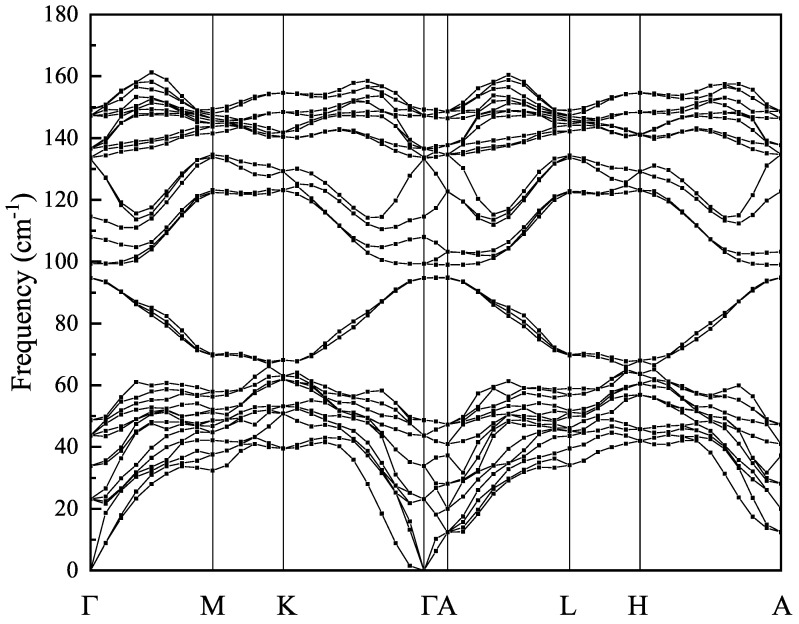
Phonon dispersion for bulk CdTe vdW crystal.

As was noted in [11], the existence of a stable inverted phase in CdTe terminated by Te atomic planes with a honeycomb arrangement of Te atoms suggests a possibility of building a bulk vdW CdTe crystal based on *inv*-2ML CdTe building blocks. Following this suggestion, we built a bulk vdW CdTe crystal and verified its thermodynamical stability by calculating a phonon dispersion spectrum (shown in Figure 6). One can see that the spectrum contains no imaginary modes, clearly demonstrating that the bulk vdW CdTe crystal is thermodynamically stable. The layered vdW CdTe crystal built from vdW blocks (1vdW = *inv*-2ML), as crystal forming elements, has the symmetry of the space group R$\bar{3}$m (Schonflies name D${}_{3d}^{5}$), belongs to the rhombohedral (trigonal) system and has a hexagonal unit cell with cell parameters a = b = 4.628 Å, c = 20.693 Å, α = β = 90${}^{\circ }$, γ = 120${}^{\circ }$. The unit cell of a vdW CdTe crystal consists of 3 vdW blocks, the atoms of which form covalent bonds, and the blocks themselves are held together by weak van der Waals forces and separated from each other by a van der Waals gap of 2.87 Å.

Summarizing our results for *zb*-nML (*n* = 1–4), *n*vdW (*n* = 1–3) and bulk solids, one can argue that all these slabs demonstrate direct band gaps at the Γ-point but different trends for the band gap variation. Thus, in Figure 5 one can see for *zb*-nML structure (*n* = 1–4) a monotonic decline of the band gap values, stronger from *n* = 1 to *n* = 2, and weaker from *n* = 2 to *n* = 3 and near-zero values for *n* = 3 and *n* = 4. For *n*vdW slabs there is a significant decline with an increase in the number of vdW blocks, however not to zero. Also the larger band gap values for vdW slabs than that for *zb* slabs are observed. The SOC inclusion leads to insignificant changes (about 0.1 eV) of the band gap values for all *n*vdW, *zb*-1ML, *zb*-2ML slabs and to the negligible changes for *zb*-3ML and *zb*-4ML (see Figure 2).

Concluding the analysis of the thickness dependencies of the band gaps, it is worth mentioning that when reducing the size of a semiconductor to nanometers one would expect an increase of the band gap due to quantum size effects [38,39,40]. This, indeed, has been observed for the structures based on vdW blocks and for ultrathin (1–2 ML) *zb*- slabs. For thicker (3–4 ML) *zb*- slabs the band gap unexpectedely decreased almost to zero. We believe this is caused by the presence of dangling bonds and surface dipoles, associated with the cations and anions of the opposite basal planes of the *zb*-2ML CdTe slab. The existence of permanent dipoles apparently leads to a decrease of calculated DFT-band gaps. In particular, this is indirectly confirmed by the fact that for the 1vdW (*inv*-2ML) CdTe slab, which does not have any permanent dipoles, the DFT-band gap in present calculations which was found to be an order of magnitude larger than that for the *zb*-2ML CdTe slab.

To summarize the discussion of the band gap variation in the *zb*-nML and *n*vdW CdTe slabs, we note that all calculated in the present DFT study band gap values are strongly underestimated due to the incomplete exclusion of the electron self-interaction within the GGA approximation for the XC-functional [41,42]. Therefore the experimental values are expected to be larger than the calculated values. More accurate calculations of the band gap can be achieved in one of four ways, namely, by using (i) the Tran-Blaha XC-functional [16] or (ii) different hybrid XC-functionals, (iii) nonlocal self-interaction correction (SIC) [41,42] or, finally, (iv) the many-body perturbation theory method (GW-method). However, this is beyond the scope of the present work whose aim was to study trends in their thickness dependences rather than absolute values.

**Figure 7:**
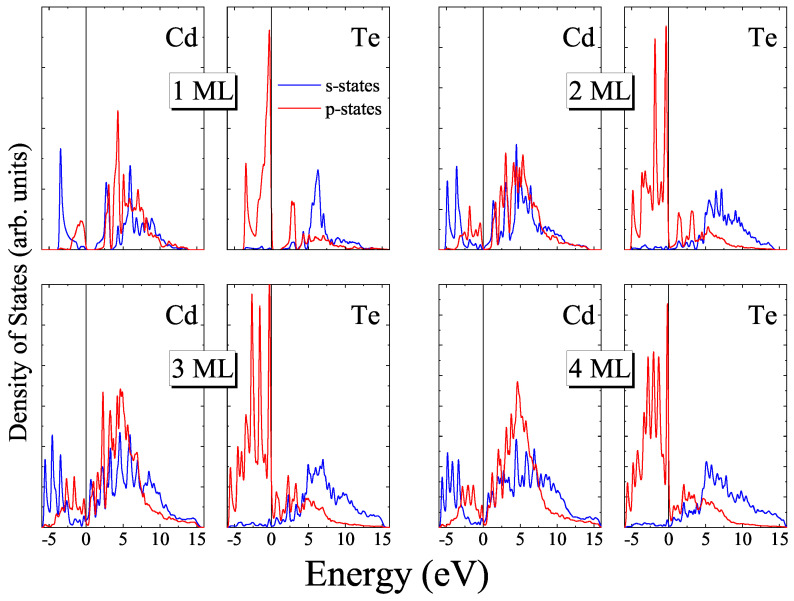
Partial densities of states for *zb*-nML (*n* = 1−4) CdTe.

In addition to the band structures, partial densities of states were calculated for all the slabs under study. The calculated PDOS for *zb*-nML CdTe slabs demonstrate that the top of the valence band is formed by p- states of Te while the conduction band consists of s- and p- states of Cd and s- states of Te. It is interesting to note that for all *zb*-nML (*n* = 1–4) slabs the number of peaks near the top of the valence band coincides with the number of layers (see Figure 7).

**Figure 8:**
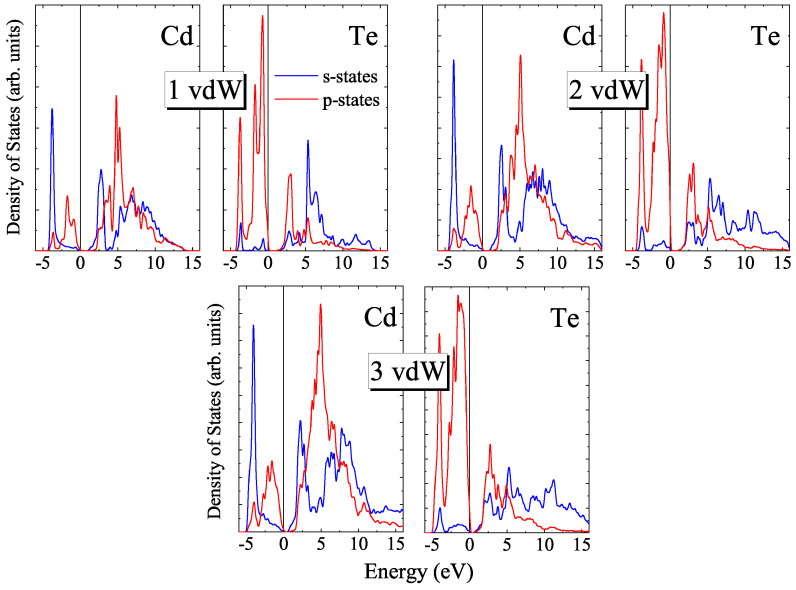
Partial densities of states for CdTe vdW blocks.

The calculated PDOS for *n*vdW CdTe slabs are shown in Figure 8. One can see that for the slabs of the inverted phase the top of the valence band is formed by p- states of Te as also is the case for the zinc-blende phase. The bottom of the conduction band is formed by both s- and p- states of Cd.

**Figure 9:**
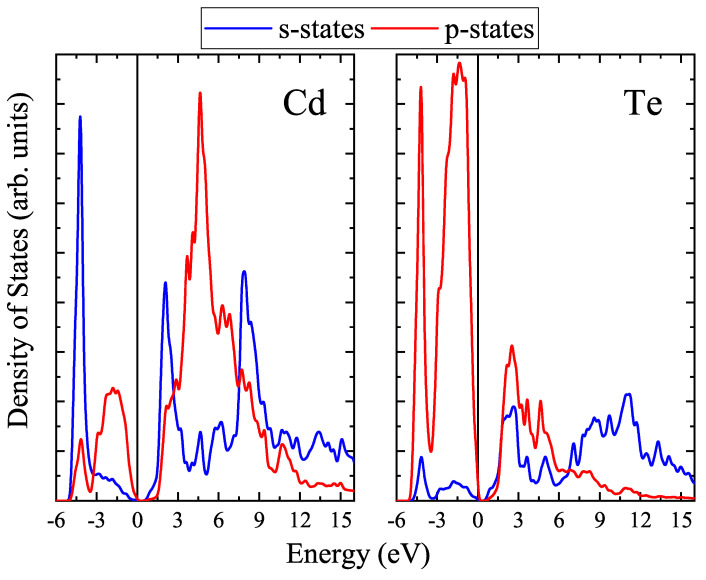
Partial densities of states for bulk CdTe vdW crystal.

PDOS of the bulk vdW CdTe crystal is shown in Figure 9. One can see that the top of the valence band consists mainly of 5p-states of Te atoms, the contribution of 5s-states for both types of atoms being negligible. The bottom of the conduction band is determined by 5s-states of Cd and hybridized 5s- and 5p-states of Te. The genealogy of the states for the bulk vdW CdTe was found to be similar to that of the bulk *zb*-CdTe reported at [43].

The main difference in the band structures of the bulk *zb*-CdTe and the bulk vdW CdTe crystals is in the character of the band gap (see Figure 1, Figure 2, Figure 3 and Figure 4). While the bulk *zb*-CdTe crystal is a semiconductor with a direct band gap of 0.575 eV at the Γ-point without SOC (0.442 eV with SOC) for PBE XC-functional and 0.680 eV without SOC (0.517 eV with SOC) for PBEsol XC-functional, the bulk vdW CdTe crystal has an indirect band gap of 0.537 eV without SOC (0.560 eV with SOC) for PBE XC-functional with D2-Grimme vdW corrections.

## 4. Summary

In the present work the thickness dependence of the band gap values for thin CdTe slabs with different layer stackings was studied by the DFT method using PBE and PBEsol XC-functionals taking into account the vdW correction in the form of D2-Grimme for the case of vdW slabs. It was shown that slabs with a thickness of one to several monolayers of both phases are suitable for fabricating structures with a variable band gap. Based on vdW blocks a bulk vdW CdTe crystal was built. The thermodynamical stability of the thus constructed bulk vdW CdTe crystal was verified by DFT calculation of the phonon dispersion spectrum. Finally, the electronic structure of the constructed vdW CdTe crystal was calculated and analyzed. Our results provide a deeper knowledge into the structure and properties of 3D semiconductors in the monolayer-thick limit and will serve to develop novel electronic and optoelectronic devices including flexible and transparent electronics.

## Figures and Tables

**Figure A4:**
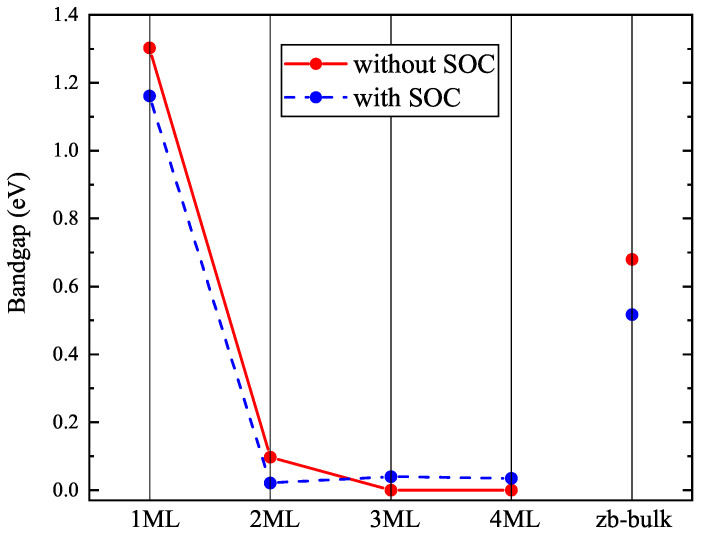
Thickness dependence of the PBEsol band gap value of *zb*-CdTe and *inv*-CdTe.

## Data Availability

Additional data can obtained from the authors upon reasonable request.

## Abbreviations

The following abbreviations are used in this manuscript:

2D	two-dimensional
3D	three-dimensional
ML	monolayer
DFT	density functional theory
DFPT	density functional perturbation theory
XC	exchange-correlation
SOC	spin-orbit coupling
GGA	generalized gradient approximation
PBE	Perdew-Burke-Ernzerhof
PBEsol	Perdew-Burke-Ernzerhof for solids
ONCV	optimized norm-conserving Vanderbilt
FP-LAPW	full-potential linearized augmented plane wave
SR	scalar-relativistic
FR	fully relativistic
vdW	van der Waals
SIC	self-interaction correction
PAW	projected augmented wave
CASTEP	CAmbridge Serial Total Energy Package
BFGS	Broyden-Fletcher-Goldfarb-Shanno
BZ	Brillouin zone
DOS	density of states
PDOS	partial density of states
